# Influence of a chronic beta-blocker therapy on perioperative opioid consumption – a post hoc secondary analysis

**DOI:** 10.1186/s12871-024-02456-2

**Published:** 2024-02-27

**Authors:** Ralf F. Trauzeddel, Luisa M. Rothe, Michael Nordine, Lukas Dehé, Kathrin Scholtz, Claudia Spies, Daniel Hadzidiakos, Georg Winterer, Friedrich Borchers, Jochen Kruppa, Sascha Treskatsch

**Affiliations:** 1 Department of Anesthesiology and Intensive Care Medicine, Campus Benjamin Franklin, Charité - Universitätsmedizin Berlin, Corporate Member of Freie Universität and Humboldt Universität zu Berlin, Hindenburgdamm 30, Berlin, 12203 Germany; 2 IS Global Campus Cliníc Rosselló, Barcelona Institute for Global Health, 132, 7è, Barcelona, 08036 Spain; 3 Department of Anesthesiology, Intensive Care Medicine, and Pain Therapy, University Hospital Frankfurt, Goethe University Frankfurt, Frankfurt, Germany; 4 Department of Anesthesiology and Intensive Care Medicine, Charité - Universitätsmedizin Berlin, Corporate Member of Freie Universität and Humboldt Universität zu Berlin, Augustenburger Platz 1, Berlin, 13353 Germany; 5 Hochschule Osnabrück, University of Applied Sciences, Osnabrück, Germany

**Keywords:** Beta-blocker therapy, Opioids, Cardiovascular, Noncardiac surgery

## Abstract

**Background:**

Beta-blocker (BB) therapy plays a central role in the treatment of cardiovascular diseases. An increasing number of patients with cardiovascular diseases undergoe noncardiac surgery, where opioids are an integral part of the anesthesiological management. There is evidence to suggest that short-term intravenous BB therapy may influence perioperative opioid requirements due to an assumed cross-talk between G-protein coupled beta-adrenergic and opioid receptors. Whether chronic BB therapy could also have an influence on perioperative opioid requirements is unclear.

**Methods:**

A post hoc analysis of prospectively collected data from a multicenter observational (BioCog) study was performed. Inclusion criteria consisted of elderly patients (≥ 65 years) undergoing elective noncardiac surgery as well as total intravenous general anesthesia without the use of regional anesthesia and duration of anesthesia ≥ 60 min. Two groups were defined: patients with and without BB in their regular preopreative medication. The administered opioids were converted to their respective morphine equivalent doses. Multiple regression analysis was performed using the morphine-index to identify independent predictors.

**Results:**

A total of 747 patients were included in the BioCog study in the study center Berlin. 106 patients fulfilled the inclusion criteria. Of these, 37 were on chronic BB. The latter were preoperatively significantly more likely to have arterial hypertension (94.6%), chronic renal failure (27%) and hyperlipoproteinemia (51.4%) compared to patients without BB. Both groups did not differ in terms of cumulative perioperative morphine equivalent dose (230.9 (BB group) vs. 214.8 mg (Non-BB group)). Predictive factors for increased morphine-index were older age, male sex, longer duration of anesthesia and surgery of the trunk. In a model with logarithmised morphine index, only gender (female) and duration of anesthesia remained predictive factors.

**Conclusions:**

Chronic BB therapy was not associated with a reduced perioperative opioid consumption.

**Trial registration:**

This study was registered at ClinicalTrials.gov (NCT02265263) on the 15.10.2014 with the principal investigator being Univ.-Prof. Dr. med. Claudia Spies.

**Supplementary Information:**

The online version contains supplementary material available at 10.1186/s12871-024-02456-2.

## Background

Chronic beta-blocker (BB) therapy plays a central role in the treatment of cardiovascular disease, which is one of the leading causes of morbidity and mortality in western societies. BBs are indicated in the treatment of chronic heart failure [1] in supraventricular tachyarrythmias, coronary artery disease as well as post myocardial infarction [2]. Due to their competitive antagonism at G-protein coupled beta-adrenoceptors, they suppress sympathetic innervation and thus have negative chronotropic, dromotropic, inotropic and lusitropic effects, thereby reducing heart rate and cardiac workload. BB treatment in the aforementioned cardiovascular diseases therefore results in a reduction of mortality [3]. In this context, it is worth mentioning that an increasing number of patients with cardiovascular risk factors with ongoing BB treatment are scheduled for noncardiac surgery [4].

Opioids play an integral role in anesthesiological and analgesia management. They are considered the cornerstone of general anesthesia in surgical patients presenting with preexisting cardiovascular diseases. Experimental data has demonstrated that opioids may migitate the cardioprotective effects during myocardial ischemic situations [5, 6]. Opioid receptors, also members of the G-protein-coupled receptor superfamily, are co-expressed with beta-adrenergic receptors [7]. Interestingly, there seems to be an interaction between the adrenergic and opioidergic system in the form of a cross-talk between both G-protein coupled receptors [8]. Moreover, there have been some meta-analyses showing perioperative opioid sparing effects of a short-term treatment of the short-acting BB Esmolol [9–11]. Its application resulted in a reduced postoperative pain intensity and postoperative nausea and vomiting (PONV) [10, 11]. In contrast, there is currently little understanding of the impact of a long-term “chronic” BB therapy on perioperative opioid use.

Thus, this retrospective study aimed to examine whether a chronic BB therapy is associated with a lower perioperative opioid consumption and improved perioperative outcomes with regard to perioperative parameters.

## Methods

### Study design

This study was designed as a post hoc analysis based on the study “Biomarker Development for postoperative Cognitive Impairment in the Elderly (BioCog)”, a multicenter, prospective clinical observational study with the aim to develop biomarkers to assess the risk and predict the occurrence of postoperative delirium (POD) and postoperative cognitive dysfunction (POCD) [12]. All procedures involving humans were in accordance with the ethical standards of the institutional research committee and with the 1964 Declaration of Helsinki and its later amendments. The study was approved by the local ethics committee at Charité – Universitätsmedizin Berlin (EA 2/092/14) on the 31.07.2014 and registered at ClinicalTrials.gov (NCT02265263) on the 15.10.2014 with the principal investigator being Univ.-Prof. Dr. med. Claudia Spies. Informed written consent was obtained from all patients. Data collection for the entire BioCog study took place between October 2014 and June 2019 at the Department of Anesthesiology and Intensive Care Medicine at Campus Charité Mitte and Campus Virchow-Klinikum, Charité – Universitätsmedizin Berlin, Germany, and the Department of Intensive Care Medicine at the University Medical Center Utrecht, Netherlands. We adhered to the CONSORT guidelines and the STROBE checklist.

### Study population

The following patients were screened for inclusion in the post hoc secondary analysis to examine whether a chronic BB therapy had an influence on perioperative opioid consumption: elective non-cardiac surgery (general, thoracic, orthopedic, trauma, gynecologic, urologic, ear nose, throat, neurosurgical, dermatologic) with an anesthesia duration of ≥ 60 min, total intravenous general anesthesia without additional regional anesthesia. Only BioCog study participants from Berlin, Germany, were included in the secondary analysis. Patients, who fulfilled the inclusion criteria, were divided into two groups, whether they received a chronic BB therapy as a regular medication at the time of study inclusion or not, and compared to each other. Additionally, we exluded patients with chronic pain from our post hoc analysis to harmonise the study cohort. In the main study, patients were advised to take their BB on the morning of surgery and to resume BB treatment as quickly as possible postoperatively.

### Study protocol

The study protocol has been previously described [12]. Preoperative data were collected from the preoperative visit as well as the individual patient file and included age, gender, weight, Body Mass Index (BMI), American Society of Anesthesiologists Physical Status (ASA PS), type of planned surgery, consumption of alcohol and/or nicotine, preexisting medical conditions and regular medication intake. Perioperative data were extracted from the electronic patient data management system (COPRA6®). This included the duration of anesthesia, administered medications, cumulative opioid application including all opioids administered from the beginning of anesthesia until the end of recovery room treatment, length of stay in the recovery room, PONV, incidence of intolerable pain and rate of admission to the intensive care unit (ICU)/postanaesthesia care unit (PACU). Postoperative data included length of hospital stay and rate of complications based on the Clavien-Dindo-Classification [13, 14]. For the ease of use, complications were divided into the following categories: 1) no complication, 2) complications (without death), 3) death. The follow-up period was from the beginning of treatment in the recovery room or ICU/PACU until discharge from hospital or death of the patient.

Postoperative pain was examined using the Numeric Rating Scale (NRS), Behavioral Pain Scale (BPS), BPS non intubated (BPS-NI), or the Critical-Care Pain Observation Tool (CPOT) [15–17]. For an easier description of postoperative pain, multiple scores were compiled into one cumulative pain score for the BioCog study. This score only differentiates between tolerable and intolerable pain until the 7. postoperative day. The former was defined as a pain with NRS > 4 or BPS/BPS NI > 5 or COPT > 2 in one of the postoperative visits, which were undertaken twice daily in the morning and evening until the 7. postoperative day.

### Opioid and beta-blocker equivalents

Opioids identified from anesthesia protocols included Fentanyl, Remifentanil, Morphine and Piritramid. Perioperative administered opioids were converted to their respective morphine equivalent dose according to their therapeutic potency [18]. For better comparability, the morphine index was also determined, defined as the cumulative perioperative morphine consumption divided by BMI. For comparison of the different BB, the value of the achieved target dose in percent was calculated, according to the guideline of the European Society of Cardiology (ESC) for the diagnosis and treatment of acute and chronic heart failure of 2016 [19].

### Statistical analysis

All statistical analyses are exploratory in nature and not considered confirmatory. Statistical analysis was done using SPSS^®^ Statistics 25 (SPSS, Inc., Chicago, IL). Graphics were created using both SPSS and Excel. All binary and categorical variables are reported as absolute and relative frequencies, continuous non-normally distributed variables are listed as median with interquartile range (IQR). Normal distribution was tested using histograms and the Kolmogorov-Smirnov test. Testing for differences between two independent groups was done using Mann-Whitney U test for continuous non-normally distributed variables and Pearson chi-square test for categorical non-normally distributed variables.

A simple linear regression analysis was used to test whether beta-blockade or the dose of BB therapy had an effect on perioperative opioid consumption. Multiple regression analysis was used to identify risk factors for predicting an increased or decreased morphine index. Subsequently, a normal distribution of the data used was obtained by regressing the morphine index, which allowed further precision of the confounder adjustment. The individual significant predictors were then graphically represented using bivariate regression analysis and grouped boxplots.

## Results

### Patient characteristics

**Fig. 1:**
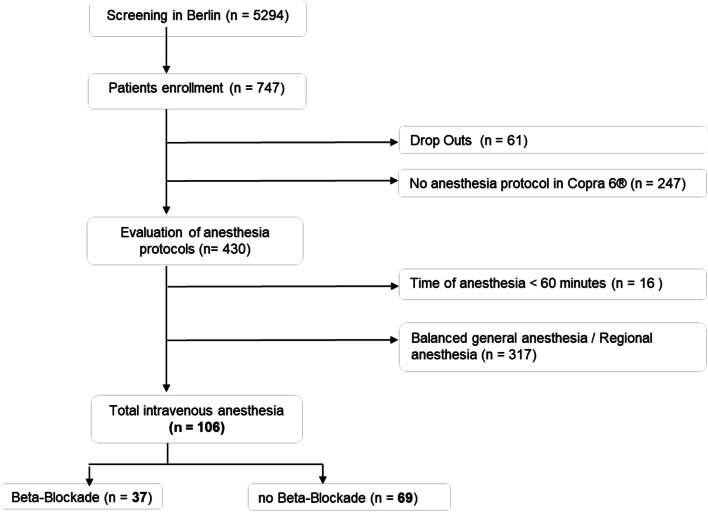
Patient enrollment

In total, 747 patients were recruited for inclusion in the BioCog study in Berlin. Thereof, 247 patients were exluded for post hoc analysis, as their respective anesthesia protocol was recorded with an older version of the patient data management system where an exact calculation of the perfusor-controlled opioid administration, as is the case with Remifentanil, was not possible. Of the remaining 439 analyzed protocols, 106 patients fulfilled the inclusion criteria of which 37 patients had a long-term BB therapy (Fig. 1). Baseline characteristics were similar between groups (Table 1).

**Table 1:** Baseline characteristics of the study population

	**n**	**Beta-Blockade (*n* = 37)**	**No Beta-Blockade (*n* = 69)**	***P*-value**
Age (years)	106	73 (69.5–77.0)	72 (68.0–76.0)	0.393^1^
Gender: female	106	22 (59.5%)	40 (58.0%)	0.882^2^
Body weight (kg)	106	72 (66–80)	78 (67–84)	0.251^1^
Body height (cm)	106	165 (160–170)	169 (160–177)	0.245^1^
BMI (kg/m^2^)	106	26.1 (24.1–27.6)	25.9 (23.8–28.6)	0.923^1^
ASA-PS:	106			0.228^2^
ASA I-II		22 (59.9%)	49 (71%)	
ASA III-IV		15 (40.5%)	20 (29.0%)	
Type of surgery:	106			0.787^2^
Intracranial		2 (5.4%)	3 (4.3%)	
Thoracic/abdominal/pelvic		10 (27.0%)	15 (21.7%)	
Peripheral		25 (67.6%)	51 (73.9%)	

Median with 25^th^ and 75^th^ percentile or absolute and relative frequencies (indicated by a frequency sign)

*n* Number of patients, *ASA-PS* American Society of Anesthesiologists Physical Status Classification, *BMI* Body Mass Index

^1^Mann-Whitney-U-Test

^2^Chi-Square-Test

Preexisting medical conditions are displayed in Table 2. Patients with chronic BB therapy presented with significantly more arterial hypertension, chronic renal failure and hyperlipoproteinemia.

**Table 2:** Prevalence of preexisting medical conditions of the study population

	**n**	**Beta-Blockade (*n* = 37)**	**No Beta-Blockade (*n* = 69)**	***P*-value**
Arterial hypertension	103	35 (94.6%) (*n* = 37)	40 (60.6%) (*n* = 66)	< 0.001^2^
Coronary artery disease	102	8 (22.2%) (*n* = 36)	7 (10.6%) (*n* = 66)	0.113^2^
Peripheral arterial disease	106	4 (10.8%)	2 (2.9%)	0.093^2^
Ischemic stroke/TIA	101	0	3 (4.7%)	0.181^2^
Atrial fibrillation	106	7 (18.9%)	5 (7.2%)	0.071^2^
Chronic kidney injury	106	10 (27.0%)	6 (8.7%)	0.012^2^
COPD/Asthma	106	6 (16.2%)	5 (7.2%)	0.149^2^
Diabetes mellitus	103	11 (29.7%) (*n* = 37)	14 (21.2%) (*n* = 66)	0.333^2^
Hyperlipo-proteinemia	106	19 (51.4%)	15 (21.7%	0.002^2^
Smoking	103	7 (20%) (*n* = 35)	6 (8.8%) (*n* = 68)	0.106^2^
Package years	87	0 (0–7.5) (*n* = 27)	0 (0–11.9) (*n* = 60)	0.698^1^
Alcohol consumption:	96	*n* = 34	*n* = 62	0.238^2^
Non-harmful	91	31 (91.2%)	60 (96.8%)	
Harmful	5	3 (8.8%)	2 (3.2%)	

Median with 25^th^ and 75^th^ percentile or absolute and relative frequencies (indicated by a frequency sign)

*n* Number of patients, *TIA* Transient ischemic attack, *COPD* Chronic obstructive pulmonary disease

^1^Mann-Whitney-U-Test

^2^Chi-Square-Test

### Perioperative data

Data of the perioperative phase and incidence of complications are shown in Tables 3 and 4, respectively. Patients on chronic BB therapy did not differ significantly from patients without BB therapy regarding the cumulative total opioid dose during surgery. Similarly, median anesthesia times were similarly distributed. There were no differences in the amount of administered blood products, catecholamines, antihypertensive agents and atropine. Postoperatively, no differences concerning intolerable pain, PONV as well as recovery room length of stay between both groups were detected. Admission to ICU or PACU as well as length of stay in the ICU or PACU (BB: 0 days (0–0.1); no BB: 0 days, *p* = 0.608) in median and hospital length of stay (BB: 6 (3.5–9) days; no BB: 6 (3.5–9) days, *p* = 0.828) in median did not differ between both groups. The incidence of postoperative complications are given in Table 4, revealing no significant differences between groups.

**Table 3:** Perioperative data of the study population

	**n**	**Beta-Blockade (*n* = 37)**	**No Beta-Blockade (*n* = 69)**	***P*-value**
Total cumulative perioperative morphine equivalent dose (mg)	106	230.9 (50.0–446.5)	214.8 (44.3–431.5)	0.910^1^
Duration of anesthesia (min)	106	136 (91–231)	124 (89–207)	0.480^1^
Perioperative application of catecholamines	106	33 (89.2%)	56 (81.2%)	0.283^2^
Perioperative application of antihypertensive medication	106	7 (18.9%)	8 (11.6%)	0.302^2^
Atropin	106	5 (13.5%)	9 (13.0%)	0.946^2^
Blood products	106	2 (5.4%)	2 (2.9%)	0.519^2^
Incidence PONV	106	8 (21.6%)	10 (14.5%)	0.351^2^
Incidence non- tolerable pain recovery room	106	15 (40.5%)	24 (34.8%)	0.558^2^
Application of opioids	106	21 (56.8%)	31 (44.9%)	0.246^2^
Total cumulative morphine equivalent dose recovery room (mg)	106	2.1 (0–7.35)	0 (0–7)	0.371^1^
Recovery room length of stay (min)	91	150 (96–207)	116.5 (84–169.25)	0.342^1^
Admission to ICU/PACU	106	10 (27%)	16 (23.2%)	0.661^2^
Readmission to hospital within 30 days postoperative	106	6 (16.2%)	6 (8.7%)	0.244^2^

Median with 25^th^ and 75^th^ percentile or absolute and relative frequencies (indicated by a frequency sign)

*n* Number of patients, *ICU* Intensive care unit, *PACU* Postanesthesia care unit

^1^Mann-Whitney-U-Test

^2^Chi-Square-Test

**Table 4:** Incidence of postoperative complications

	**n**	**Beta-Blockade (*n* = 37)**	**No Beta-Blockade (*n* = 69)**	***P*-value**
Incidence postoperative total complications	106	19 (51.4%)	37 (53.6%)	0.823^2^
Incidence postoperative delirium	106	8 (21.6%)	17 (24.6%)	0.727^2^
Incidence postoperative non-tolerable pain	104	14 (37.8%) (*n* = 37)	21 (31.3%) (*n* = 67)	0.502^2^
Incidence postoperative arrhythmia	106	2 (5.4%)	2 (2.9%)	0.519^2^
Incidence postoperative ischemic complications	106	2 (5.4%)	1 (1.4%)	0.242^2^
Incidence postoperative wound infections	106	2 (5.4%)	2 (2.9%)	0.519^2^

Median with 25^th^ and 75^th^ percentile or absolute and relative frequencies (indicated by a frequency sign)

*n* Number of patients, *ICU* Intensive care unit, *PACU* Postanesthesia care unit

^1^Mann-Whitney-U-Test; ^2^Chi-Square-Test

### Influences on perioperative opioid consumption

There was no correlation between doses of chronic BB therapy and increasing morphine equivalents (Supplementary Fig. 1).

Using multiple regression analyses independent predictors for the amount of perioperative opioid consumption, expressed by the morphine index, were examined (Table 5). Chronic BB therapy had no influence on the opioid consumption, whereas age, gender, anesthesia duration as well as type of surgery had. Their respective influence on the morphine index is given by the regression coefficient. Regarding age, each year yielded an increase of the morphine index by 0.547 mg/BMI, whereas female gender leads to a reduction of the morphine index by 9.104 mg/BMI (Table 5). Intracranial surgery as well as thoracic, abdominal and pelvic surgery were associated with an increased morphine index compared to surgery involving the extremities.

**Table 5:** Multiple regression analysis of morphine index (*n* = 106)

	**B (95% CI)**	**SE**	***P*-value**
Age (years)	0.547 (0.09;1.00)	0.229	0.019
Gender (female)	-9.104 (-13.73;-4.48)	2.333	0.000
Chronic Beta-blocker therapy (no/yes)	-1.052 (-5.68;3.57)	2.331	0.653
Duration of anesthesia	0.090 (0.07;0.11)	0.010	0.000
Type of surgery	5.086 (0.77;9.40)	2.175	0.021

Regression coefficient

*95% CI* 95% Confidence Interval, *SE* Standard Error of Mean

## Discussion

This retrospective post hoc analysis of the BioCog study was not able to detect an influence of chronic BB therapy on perioperative opioid consumption amongst noncardiac surgical patients undergoing general anesthesia. Age, gender, duration of anesthesia and type of surgery were confirmed to be of predictive value concerning an increased consumption of perioperative opioids.

Recently, there have been several studies indicating that short-term perioperative BB therapy with Esmolol can reduce perioperative opioid consumption [9, 10, 20–23], which cannot be translated to our “chronic” setting. Possible explanations could be that compared to patients on chronic BB therapy, these patients were younger and did not exhibit cardiovascular comorbidities or other similar risk factors. It is known that reactivity of beta-adrenergic receptors decreases with increasing age, termed beta-adrenoceptor desensibilisation, as demonstrated in animal and human studies. Beta-adrenoceptor desensibilisation is theorisized to be due to phosphorylation of receptor structures in agonist-receptor binding states leading to a decrease of receptor density and subsequent internalization, which has also been described amongst heart failure [24–26]. This further leads to a reduced autonomic modulation of the cardiac system during physical activity [27] and possibly to a diminished perioperative sympathetic reaction to pain. This change in receptor structure and function is one of the main causes of chronic BB therapy to counteract internalization.

Nonetheless, Starr et al. showed that chronic BB therapy was associated with a lower prescription of postoperative opioids within the first 30 days of surgery in a large retrospective cohort study of American veterans undergoing total knee arthroplasty implantation [28]. Furthermore, selective as well as non-selective BB were associated with a reduced postoperative morphine equivalent dose up to 30 days. However, compared to our study, Starr et al. only examined the postoperative period beginning from the first postoperative day analysing oral opioid medication. Moreover, chronic BB therapy was assumed if patients were prescribed BB within 90 days before surgery or during hospitalisation and/or within the first 90 days postoperatively. With regard to our study, we are not able to make any statement about the duration of the use of BB therapy preoperatively. Additionally, postoperative prescription rate concerning opioids differ between the United States and Germany, which may additionally explain the divergent study results [29].

A possible reason for the potential of a reduced perioperative consumption under BB therapy might be the concept of a cross-talk between beta-adrenergic and opioid receptors. In animal models, these receptors are co-expressed at the sarcolemma of cardiomyocytes [30–32]. This might explain that Esmolol was shown to diminish sympathetic excitation perioperatively caused by PONV in the literature [9–11]. However, it is unclear if these effects could also apply to a chronic BB therapy.

Additionally, stimulation of δ- and κ-opioidreceptors not only mediated cardioprotective effects against ischemic and hypoxia-induced injury in animal models, but also limited the positive inotropic and chronotropic effects of catecholamines [8, 33–40]. A clinical transitional study showing a possible interaction between both receptors in the human body is still lacking. We were also not able show significant differences in perioperative use of catecholamines, antihypertensive drugs and cardiovascular complications with and without beta-blockade in our study. The reason for that could be the relative small sample size in the BB group. However, in a large cohort study by Ahl et al. patients presenting for emergent colonic cancer surgery also did not differ regarding perioperative complications whether they were on chronic beta-blockade or not [41].

Beyond our primary study we were able to show that several factors were associated with an increased opioid usage. One of these specific factors was gender. Gender-specific differences in pain perception have been described in the literature. Regarding postoperative pain ambiguous results were reported [42–49]. However, none of these studies is methodologically comparable to our investigation. Physiologically, gonadal steroid hormones (e.g. estradiol, testosterone) have a modulating effect on pain perception and analgesia. They can influence the pharmacokinetics and pharmacodynamics of opioids by altering absorption and distribution as well as metabolism of opioids to active and inactive metabolites [50, 51]. Oestrogens can attenuate the effects of endogenous and exogenous opioids by binding directly to opioid receptors [52].

Type of surgery was also associated with an increased perioperative morphine index, especially in abdominal surgery. Yet, the ratio of abdominal to peripheral surgery was 25:76, this uneven cohort as well as longer surgery and anesthesia duration might have obscured the results. Nevertheless, our results are in contrast to an observational study by Ekstein et al., which compared abdominal to orthopedic surgery. The authors found no differences regarding intraoperative cumulative doses of fentanyl, but higher postoperative pain scores after orthopedic surgery [53].

To our knowledge, there exist only few studies investigating perioperative opioid consumption in elderly patients up to now [54]. Pathophysiologically several mechanisms might be an explanation for the observed association between age and opioid consumption. With increasing age, a large propotion of metabolically active tissue is converted to fat leading to altered distribution volumes of lipo- and hydrophilic drugs [55]. Cardiac output decreases with a change in distribution from kidneys and liver to heart and brain [56]. Renal parenchyma and renal clearance as well as liver mass and hepatic blood flow decreases [57, 58]. All these result in a significantly lower clearance, prolonged terminal elimination half-time and higher serum concentrations of opioids in elderly compared to younger patients [59]. However, it remains unclear why more opioids were administered (or needed) by the older patients in our study. Also amongst older patients, Remifentanil was deployed more often than other opioids as part of the anesthesia plan due to its favourable effects, e.g. short-half life, organ-independent degradation, etc. As Remifentanil was calculated with an morphin equivalent of 200, it had a significant impact on the calculated morphine equivalent dose compared to other opioids.

### Limitations

There are several limitations applying to our study. Concerning its post hoc design, an adequate power analysis and sample size calculation was not performed. Secondly, chronic beta-blockade was only defined by its preoperative existence, not by its duration before surgery. We also did not record the total duration of the chronic BB treatment. Compared to other studies, different pharmacokinetics between oral intake in chronic blockade versus intravenous application in acute beta-blockade may result in varying bioavailability. Aggregation of perioperatively administered opioids into a morphine equivalent dose without taking into consideration that pharmacokinetic differences exist between different opioids must be considered the greatest limitation. We also exluded patients who received regional anesthesia and did not analyse alternate multimodal strategies for opioid reduction in our study, as these were too heterogenous between the different types of surgery and the two campuses of which data were analysed. We might also missed other confounding factors regarding analgesic therapy, e.g. influence of non-steroidal anti-inflammatory drugs, which might have been not used in patients with vascular disease, leading to a higher use in patients with BB obscuring the effect of the latter. There was no study specific protocol for intra- and postoperative pain management due to the retrospective nature of the post hoc analysis. Due to the limited sample size, it could be possible that an effect of a chronic BB therapy on opioid consumption might not have been detected in our cohort. Therefore, prospective trials with larger sample sizes are needed to investigate this question further. Finally, there was a great amount of heterogeneity regarding types of surgery included resulting in a heterogenous study group as well as different BB that were taken in the chronic BB group. We did not collect information about the exact indication for chronic BB intake as well as about the proportion of patients who complied to the recommendations concerning perioperative BB intake. We did not analyze the effect of individual risk factors on outcome as sample size was too small. Also, we cannot for sure certify the comparability of similar procedures, i.e. procedure per specialism.

## Conclusions

In our study being one of the first to examine the influence of a chronic beta-blockade on perioperative opioid consumption, we could not show an association between these two. There is a need for randomized controlled trials and adequately powered prospective studies to examine this question further before any conclusions can be made.

### Supplementary Information

**Supplementary Material 1.**

## Data Availability

The data that support the findings of this study are available on request from the corresponding author. The data are not publicly available due to German data protection laws.

## Abbreviations

ASA PS: American Society of Anesthesiologists Physical Status
BB: Beta-Blocker
BMI: Body mass index
BPS: Behavioral Pain Scale
BPS-NI: Behavioral Pain Scale non intubated
COPD: Chronic obstructive pulmonary disease
CPOT: Critical-Care Pain Observation Tool
ESC: European Society of Cardiology
ICU: Intensive care unit
IQR: Interquartile range
NRS: Numeric Rating Scale
PACU: Postanaesthesia care unit
POCD: Postoperative cognitive dysfunction
POD: Postoperative delirium
PONV: Postoperative nausea and vomiting
TIA: Transient ischemic attack
