# Supplementary material for: Influence of a chronic beta-blocker therapy on perioperative opioid consumption – a post hoc secondary analysis
Source: BMC Anesthesiol. 2024 Feb 27;24:80. doi: 10.1186/s12871-024-02456-2 (PMC10898005; doi:10.1186/s12871-024-02456-2)
Supplement: Supplementary file 1 — Supplementary Material 1. [file 12871_2024_2456_MOESM1_ESM.pdf]

Supplementary Figure 1: Bivariate regression analysis of morphine index (mg/Body mass index) and dose equivalence of beta-blockers (%).

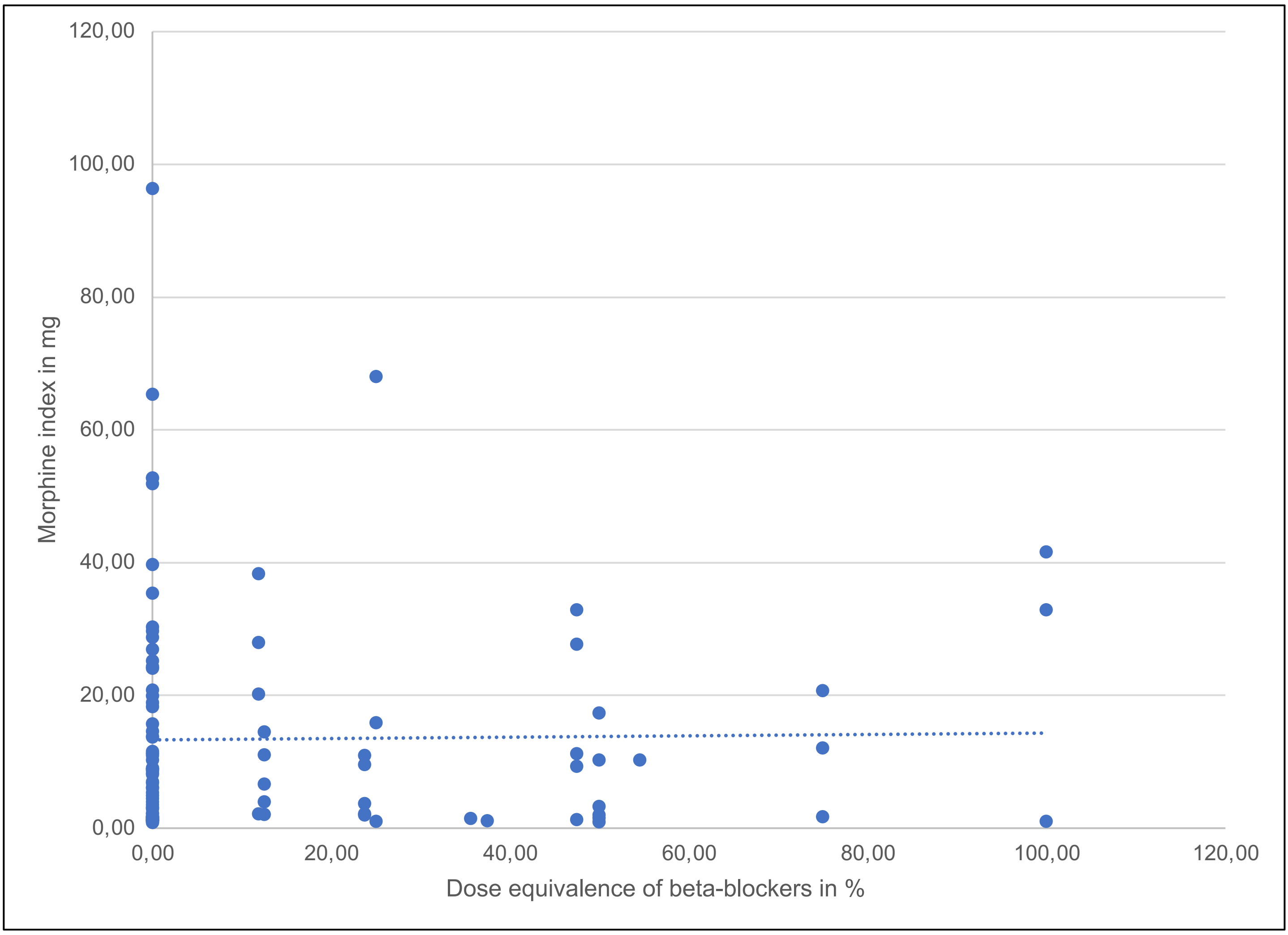

Regression equivalent for beta (95% confidence interval) = 0.010 (-0.12;0.14) (n = 106)
